# Effect of Titanium Implants Coated with Radiation-Crosslinked Collagen on Stability and Osseointegration in Rat Tibia

**DOI:** 10.3390/ma11122520

**Published:** 2018-12-11

**Authors:** Eun-Bin Bae, Ji-Hyun Yoo, Sung-In Jeong, Min-Su Kim, Youn-Mook Lim, Jong-Ju Ahn, Jin-Ju Lee, So-Hyoun Lee, Hyung-Joon Kim, Jung-Bo Huh

**Affiliations:** 1 Department of Prosthodontics, Dental Research Institute, Institute of Translational Dental Sciences, BK21 PLUS Project, School of Dentistry, Pusan National University, Yangsan 50612, Korea; 0228dmqls@hanmail.net (E.-B.B.); cyberkms01@hanmail.net (M.-S.K.); tarov0414@daum.net (J.-J.A.); ljju1112@hanmail.net (J.-J.L.); romilove7@hanmail.net (S.-H.L.); 2 Department of Oral Physiology, Dental Research Institute, Institute of Translational Dental Sciences, BK21 PLUS Project, School of Dentistry, Pusan National University, Yangsan 50612, Korea; firstlove21c@hotmail.com; 3 Advanced Radiation Technology Institute, Korea Atomic Energy Research Institute, 1266 Sinjeong-dong, Jeongeup-si, Jeollabuk-do 56212, Korea; sijeong@kaeri.re.kr (S.-I.J.); ymlim71@kaeri.re.kr (Y.-M.L.)

**Keywords:** collagen, gamma radiation, glutaraldehyde, dental implant

## Abstract

This study aimed to evaluate the titanium (Ti) implants coated with collagen type I crosslinked using gamma-irrigation or glutaraldehyde (GA). The in vitro surface observations, quantification assay, and cell studies using human mesenchymal stem cells (hMSCs) were conducted. For in vivo experiments, the implants were divided into three groups and inserted into the rat tibias: control group (non-treated Ti implant), GA group (Ti implants coated with GA-crosslinked collagen) and 25 kGy group (Ti implants coated with gamma-radiation-crosslinked collagen at dose of 25 kGy). The animals were sacrificed at 4 weeks after implantation and the tissue sections were obtained. New bone volume (mm^3^) and bone-to-implant contact (BIC, %) within the region of interest (ROI) was measured. The in vitro results showed the highest osteogenic differentiation and levels of osteogenesis-related gene expressions in the 25 kGy group without cytotoxicity. The new bone volume of GA group was significantly higher than the control (*p* < 0.05). In the result of the BIC, the 25 kGy group was significantly higher than the control (*p* < 0.05). However, there was no significant difference between the experimental groups. Within the limitations of this study, Ti implant coated with gamma-radiation-crosslinked collagen has potential utility without side effects from chemical agents.

## 1. Introduction

The close binding between the dental implant and the bone tissue affects the long-term success of the implant. In order to achieve improved osseointegration, various studies have attempted to develop the modified surface of implants [1]. Implant surface modifications, such as applying roughness, coating the biomaterials, or growth factors, can be induced to give enhanced bone regeneration, shortening the time for osseointegration and thus is able to improve the initial stability of the implant [2,3]. Among the biomaterials, collagen, which is the most abundant protein in mammals, is most widely used in clinic [4].

Collagen type I has been proven to be a highly biocompatible organic biomaterial and is easily absorbed into the host tissue after implantation [5,6,7,8]. Coating the collagen type I onto the surfaces of the implant provides an appropriate environment for osteoblasts to improve initial cell adhesion and stimulating proliferation and osteoblastic differentiation [9,10,11,12,13,14]. In animal studies, which used rabbit and goat femur models, they reported significantly increased bone growth and bone-to-implant contact (BIC) around titanium (Ti) implants treated with collagen type I [15,16]. In addition, since collagen itself acts as an osteogenic inducer for osteoconduction, it can be considered as having merits as a drug carrier [17].

It is common to use collagen after chemical crosslinking with glutaraldehyde (GA) [18,19], polyepoxide [20,21], 1,4-butanediol diglycidyl ether [22], or carbodiimide [20,23]. Among them, GA is most generally studied and used as a crosslinking agent for bioactive materials such as artificial blood vessel and bioprosthetic valve, and also used as a sterilizing agent [24,25]. The crosslinked collagen has physical and biochemical stability and reduces the antigenicity of collagen by linking the antigenic epitopes, rendering them either unapproachable to phagocytosis or indiscernible by an immune system [26]. However, most of the chemical crosslinking methods have been reported as having cytotoxicity due to the generated by-products in the crosslinking process. Besides, there are inconveniences due to the complicated processes such as removing used crosslinking agents after synthesis and re-sterilizing for removal of residual toxicity [27]. Also, in the case of GA, GA treatment is known to leave the behind cytotoxicity residues [18,26,28,29].

A gamma irradiation technique has been used for polymer crosslinking since the gamma irradiation is able to freely control the physical properties by only adjusting the irradiation dose without changing the composition of the material [30]. Crosslinking using gamma irradiation has no necessity to remove the remaining chemical additives after crosslinking since no harmful chemical crosslinking agent or initiator is used, and no need to apply heat during the reaction [31,32,33]. Furthermore, radiation crosslinking can cause a chemical reaction in a solid state or a low-temperature, and energy consumption is less than other methods due to reacting in a short time. Several studies have been used gamma irradiation on crosslinking of polymers, such as methylcellulose (MC) and carboxymethyl cellulose (CMC), and reported the stable results without toxicity [34,35]. The Fourier transform infrared (FTIR) spectra results from Rimdusit at al. [34] suggested that the crosslinking via gamma irradiation had more opportunities to form crosslink networks and induced hydrophilicity than by using chemical crosslinking.

Therefore, when the collagen is crosslinked onto the implant surfaces using gamma irradiation, it is expected that it will be possible to develop a clear and uniformly coated implant without using chemicals that are harmful to the human body. The present study was undertaken to investigate the properties of the implants coated with collagen crosslinked using gamma-irradiation compare with the coating of GA-crosslinked collagen onto the implant, and to evaluate the effects of crosslinked collagen coated implants on osseointegration in rat tibia model. The null hypothesis stated that there are no statistically significant differences in cell responses and osseointegration between gamma-radiation-crosslinked collagen coated implants using and with GA-crosslinked collagen coated implants.

## 2. Results

### 2.1. In Vitro Results

#### 2.1.1. Observations of Collagen Coated onto Ti Surface

**Figure 1:**
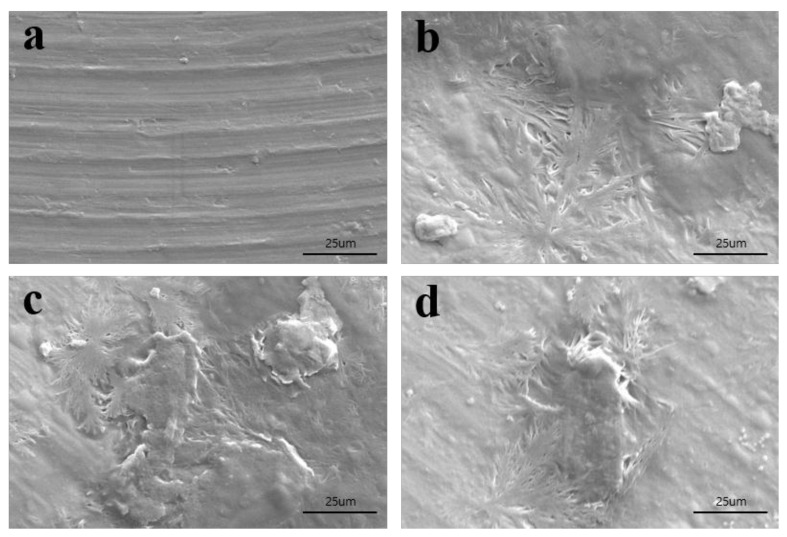
Scanning electron microscopy (SEM) images of each group: (**a**) control, (**b**) GA, (**c**) 10 kGy, and (**d**) 25 kGy (×1000 magnification).

The surface observations of non-treated Ti (Figure 1a) and collagen-coated Ti using GA (Figure 1b) or gamma irradiation (10 kGy or 25 kGy) (Figure 1c,d) was estimated using scanning electron microscopy (SEM). An almost flat surface with some shallow grooves were observed in the non-treated Ti disc (Figure 1a). In collagen-coated Ti groups, an aggregate of collagen was observed with an even coat on the Ti surface (Figure 1b–d).

#### 2.1.2. Quantification of Crosslinked Collagen Coated on Ti 

**Figure 2:**
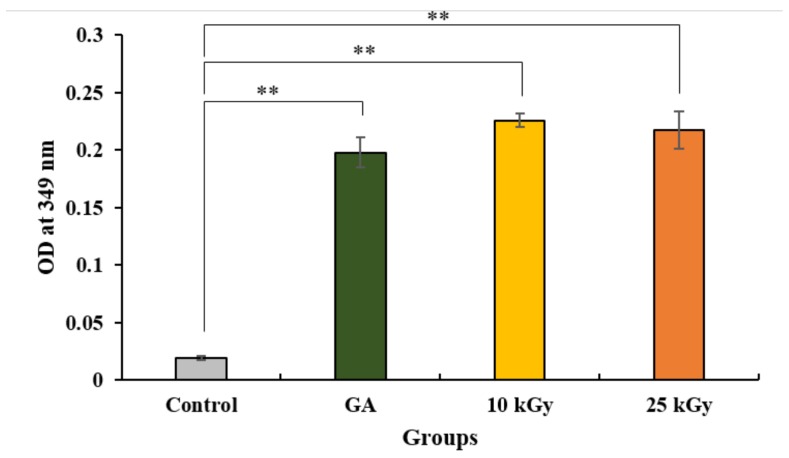
Quantification of amide groups of crosslinked collagen coated titanium implants. The symbol “*” indicates statistical significance compare to control (** *p* < 0.01).

A trinitrobenzensulonic acid (TNBS) assay was utilized to measure the crosslinked gelatin on the film. Furthermore, non-treated Ti, collagen-coated Ti using GA, collagen-coated Ti using gamma irradiation with 10 kGy and 25 kGy doses showed 0.019 ± 0.001, 0.197 ± 0.012, 0.225 ± 0.005, and 0.217 ± 0.0.016 optical density (OD) values at 349 nm, respectively (Figure 2). The amounts of collagen-coated Ti using gamma irradiation (10 kGy and 25 kGy) were found to be slightly higher values than that of the control and GA groups. We found that collagen was successfully crosslinked on the titanium implants by using glutaraldehyde or gamma irradiation.

#### 2.1.3. Cell Proliferation and Toxicity

**Figure 3:**
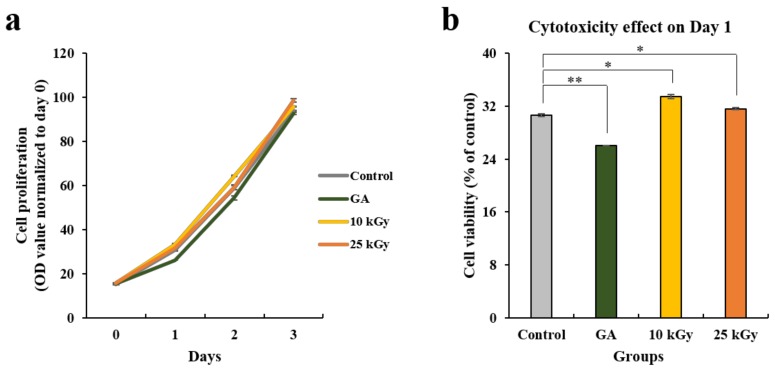
(**a**) Cell proliferation and (**b**) cytotoxicity of titanium surfaces on hMSCs. Data are mean±SD. n = 3. The symbol “*” indicates statistical significance compare to control (*p < 0.05, ** *p* < 0.01).

The proliferation rate of hMSCs cultured on the four different types of Ti discs was analyzed using an 3-[4,5-dimethylthiazol-2-yl]-2,5-diphenyl tetrazolium bromide (MTT) assay, which is a basic method for demonstrating cell proliferation. Cell numbers were analyzed spectrophotometrically at 0, 1, 2, and 3 days after cell seeding (Figure 3). Figure 3a shows the effects of the different surfaces of Ti on the proliferation of hMSCs on the indicated days of culture. There were slight differences between the cell proliferation on days 1 and 2; however, no significant difference was found over 3 days. The absorbance data of day 1 were used to evaluate toxicity of the Ti discs on the cells (Figure 3b). Ti discs irradiated using gamma rays (10 kGy and 25 kGy) were not toxic to the cells compared with the other types of discs (control and GA). The numbers of the cells cultured on gamma-irradiated Ti discs (10 kGy and 25 kGy) were found to be higher than that of GA groups on day 1.

#### 2.1.4. Analysis of Alkaline Phosphatase (ALP) Activity Assay and Alizarin Red S (ARS) Staining

**Figure 4:**
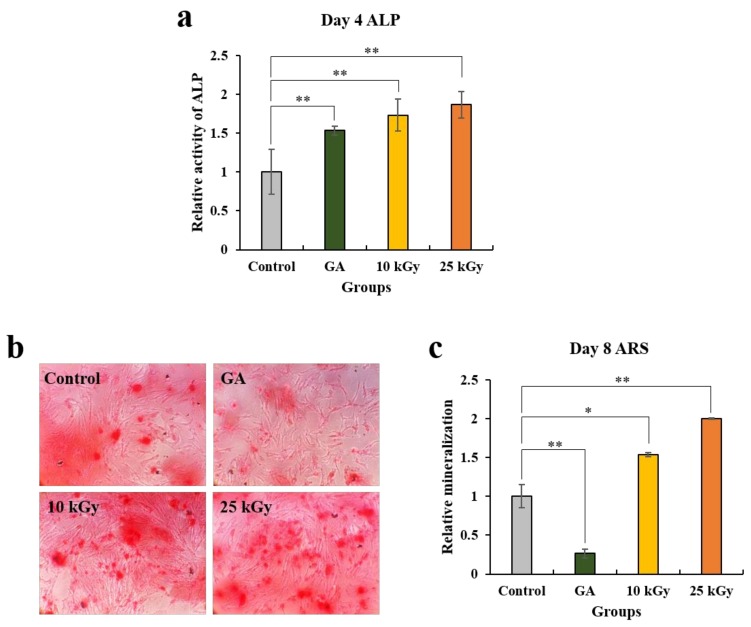
Cell osteogenic differentiation assay. (**a**) Alkaline phosphatase (ALP) activity assay and Alizarin Red S (ARS) (**b**) staining and (**c**) the quantitative analysis. Data are mean ± SD. n = 3. The symbol “*” indicates statistical significance compare to control (* *p* < 0.05, ** *p* < 0.01).

The ALP activity assay and ARS staining was conducted to analyze the effect of different kinds of Ti surfaces on the osteogenic differentiation of hMSCs (Figure 4). The cells were cultured on the four kinds of Ti discs for 8 days. On day 4, ALP activity, an early osteogenic marker, significantly increased in the cells cultured on both the 10 kGy and 25 kGy groups compared to the control (Figure 4a). In addition, the highest ALP activity was observed in the 25kGy group and the difference between the two groups (10 kGy, 25 kGy) was not obvious. Furthermore, the cells cultured on gamma-irradiated Ti discs (10 kGy and 25 kGy) showed slightly higher activity of ALP than that of the GA groups. Since the proliferation rate of each group showed no significant difference over 3 days according to the MTT assay, it is considered that the cell numbers of each group had no effect on the ALP activity. On day 8, calcium deposits significantly increased in the groups grown on the gamma-irradiated surfaces (10 kGy, 25 kGy) compared to the control according to the results of ARS staining and the quantitative analysis (Figure 4c). Moreover, the cells grown on the Ti disc (25 kGy) showed a higher differentiation rate than the cells grown on the Ti disc (10 kGy). On the contrary, ARS staining results of the cells grown onto Ti disc (GA) demonstrated a significant decrease compared to the control and the gamma-irradiated groups. Collectively, these results showed that the irradiation of gamma rays onto the surface of Ti discs had a positive effect on the regulation of osteogenic differentiation of hMSCs.

#### 2.1.5. Analysis of Real-time Polymerase Chain Reaction (PCR)

**Figure 5:**
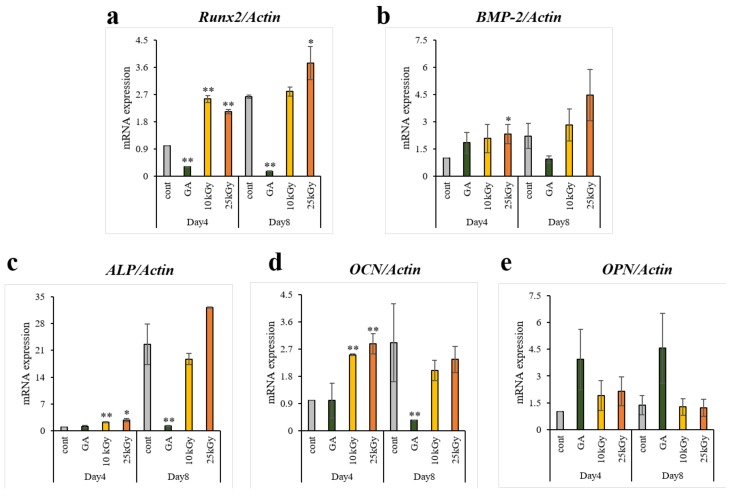
Real-time PCR analysis of hMSCs on titanium surfaces. (**a**) *Runx2*, (**b**) *BMP-2*, (**c**) *ALP*, (**d**) *OCN* and (**e**) *OPN* were selected as the osteogenic differentiation related genes. Data are mean ± SD. n = 3. The symbol “*” indicates statistical significance compare to control (* *p* < 0.05, ** *p* < 0.01).

The levels of osteogenesis-related gene expressions were analyzed by using real-time PCR (Figure 5). In comparison with the cells grown on the control and GA group, the cells grown on the Ti discs (10 kGy, 25 kGy) showed up-regulation in osteogenic transcription factor (*Runx2*) and osteogenic cytokine (bone morphogenetic protein 2 (*BMP-2*)) on both days of culture (day 4 and 8) and the both of the mRNA levels indicated a significant difference on day 4 between the control and 25 kGy group. For the expression of early osteogenic markers and an essential enzyme for ossification (ALP, osteocalcin (*OCN*), and osteopontin (*OPN*)), *ALP* and *OCN* at day 4 were significantly higher in the cells cultured on the Ti discs (10 kGy, 25 kGy) compared to the control. Conversely, the cells cultured on the Ti disc (GA) indicated lower or similar mRNA levels of osteogenic factors (*Runx2*, *BMP-2*, *ALP* and *OCN*) compared with the control except for that of *OPN*. Additionally, clear increases were observed in the gamma-irradiated groups compared with GA group in the osteogenesis related genes except for the levels of *OPN* on both days of culture and *BMP-2* on day 4. In brief, on day 4, the mRNA expression of osteogenic factors (*Runx2, BMP-2, ALP* and *OCN*) were up-regulated by the effect of gamma irradiation onto the surface of Ti discs, while the Ti disc (GA) did not affect or down-regulated the osteogenic mRNAs except for *OPN*.

### 2.2. In Vivo Results

#### 2.2.1. Clinical Findings

All rats survived during the procedure, and 12 tibia samples were harvested without issue. Any inflammation or infection was not observed at surgical sites during the healing period.

#### 2.2.2. Micro-Computed Tomography (μCT) Findings

**Figure 6:**
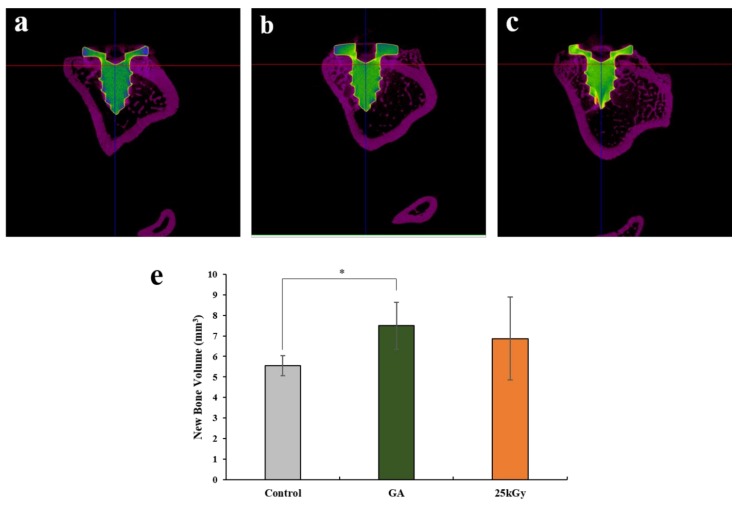
μCT images of (**a**) control, (**b**) GA and (**c**) 25 kGy. (**d**) New bone volume of each group at 4 weeks after surgery (mm^3^). The symbol “*” indicates statistical significance between the two groups (* *p* < 0.05).

The results of volumetric measurement obtained using μCT are shown in Table 1 and Figure 6. All the implants were well-positioned and maintained in each side of the tibia (Figure 6a–c). The mean ± SD of new bone volume in control, GA, and 25 kGy groups were 5.55 ± 0.48, 7.50 ± 1.14, and 6.87 ± 2.01, respectively. The new bone volume was significantly higher in the GA group compared to the control group (*p* < 0.05). There was no significant difference between the experimental groups.

#### 2.2.3. Histological Findings

**Figure 7:**
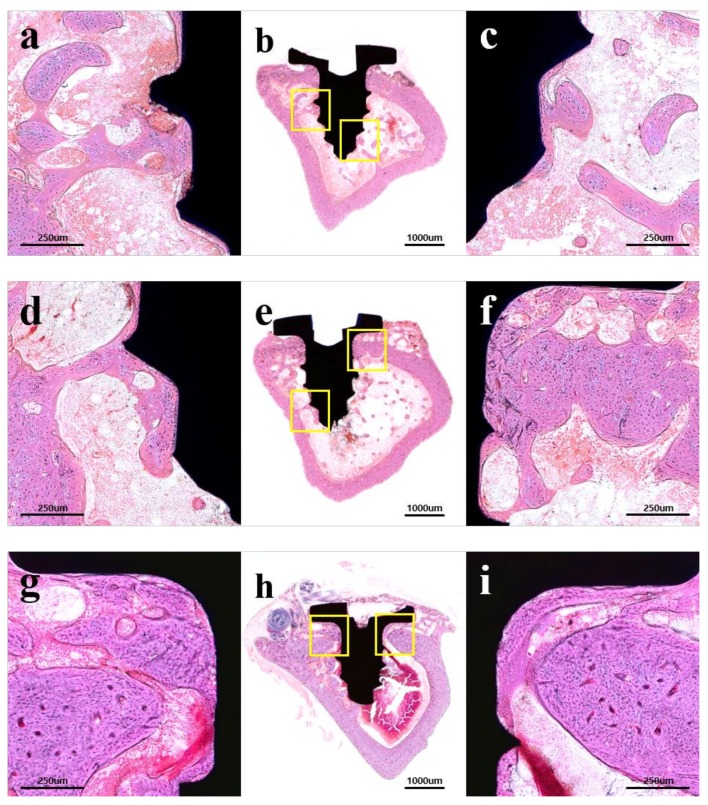
Hematoxylin-eosin (H&E) stained histological sections: (**a**–**c**) control; (**d**–**f**) GA group; (**g**–**i**) 25 kGy group; (**a**,**d**,**g**) left portion of implant; (**c**,**f**,**i**) right portion of implant; (**a**,**c**,**d**,**f**,**g**,**i**) × 100 magnification; and (**b**,**e**,**h**) × 12.5 magnification.

The results of the histological analysis are shown in Figure 7. In all groups, no abnormal findings, such as inflammatory cells, were observed. The control group showed minor new bone formation around only a few implant threads (Figure 7a–c). The experimental groups showed an enhancement in bone repair and were observed at the corticalized new bone at the bone-implant interface corresponding to the implant threads (Figure 7d–i). The 25 kGy group showed more improvement in peri-implant bone formation than other groups (Figure 7g–i).

#### 2.2.4. Histometric Findings

**Figure 8:**
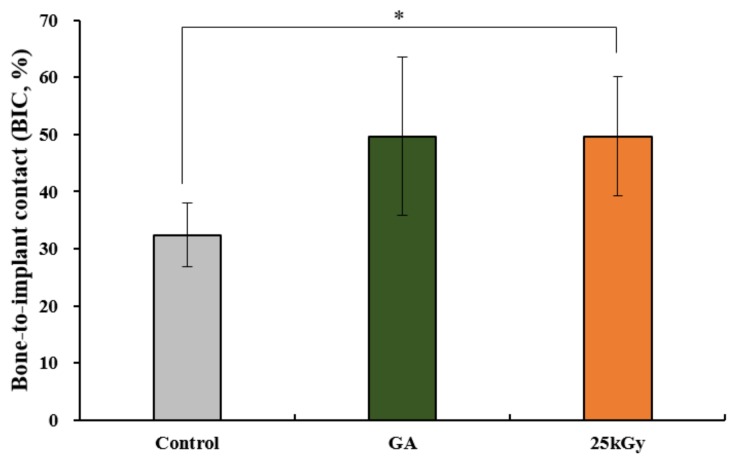
Bone-to-implant contact (BIC) of each group at 4 weeks after surgery (%). The symbol “*” indicates statistical significance between the two groups (* *p* < 0.05).

The histometric measurements are shown in Table 2 and Figure 8. The mean ± SD of bone-to-implant contact (BIC) in control, GA and 25 kGy groups were 35.21 ± 3.98, 49.66 ± 13.84, and 49.66 ± 10.47, respectively. The BIC was significantly higher in the 25 kGy group compared to the control group (*p* < 0.05). There was no significant difference between the experimental groups.

## 3. Discussion

An ideal implant surface should conduct and induce bone formation with firm osseointegration independent of the bone amount, quality, and the implant installation site [36]. With the developments of implant surface modification, studies have been actively carried out to get even better results on osseointegration and tissue responses using surface treatment with bioactive materials, especially collagen. Meanwhile, there are several drawbacks of collagen, such as immune responses by animal-derived collagen and fast degradation by the enzyme, which could limit its application [37]. To overcome these drawbacks, most collagens are used after crosslinking and the crosslinking method with chemical agents that are toxic to the cell has been most used with concern regarding the release of excess chemicals [18,19,20,21,22,23]. Recently, radiation crosslinking, which is a green and cost-effective method without any externally added chemicals, such as using gamma irradiation, electron beams, or UV have been widely used to crosslink the polymers [37,38,39,40]. Among them, gamma irradiation can homogeneously penetrate into materials and materials can be sterilized simultaneously with crosslinking; therefore, no additional sterilization is required. Zhang et al. [37] have transplanted the crosslinked collagen using gamma irradiation in vivo and confirmed the excellent biocompatibility and biodegradability control of a collagen hydrogel. Thus, this study was conducted to investigate an in vitro and in vivo comparison of Ti implants coated with collagen crosslinked using gamma irradiation and implants coated with collagen crosslinked by a typical chemical crosslinking agent: GA.

When collagen concentration or the ionization energy of the radiation is low, the crosslinking reaction may not occur properly due to the radical density of the molecular chain being lowered [41]. On the other hand, if too high a gamma dose is used, the percentage of water released from the collagen matrix will be increased, which can cause problems in the molding process [42]. The study of the influence of the absorbed dose and concentration on collagen crosslinking reported the results in increased crosslinking density at a dose of 25 kGy rather than 5 kGy with more degradation of collagen and more water squeezed out from the collagen hydrogel [37]. Furthermore, their fluorescence analysis confirmed more crosslinking points appeared in collagen crosslinked at 25kGy. In addition, the medical devices have been generally sterilized with radiation doses of 20 to 25 kGy, therefore this study used a dose of 25 kGy for sterilization and crosslinking at once.

In this study, the commercially available hMSCs were used, which are pluripotent and capable of differentiation into mesenchymal lineages such as osteoblasts, adipocytes, neurocytes, chondrocytes, etc. hMSC is the most preferred stem cells under preclinical trial and it is generally used as an osteoprogenitor cells in bone regeneration studies [43,44]. Previous studies demonstrated that the surface properties, chemistry, energy, roughness, and topography have an effect on the proliferation and differentiation of the hMSCs [45,46,47,48]. In this study, differently modified surfaces of Ti discs were tested for the response of hMSCs. The cells cultured on the four different types of Ti discs showed no significant effect on their proliferation rate. However, the cells grown on gamma-irradiated Ti discs (10 kGy, 25 kGy) were up-regulated in their osteogenic differentiation and bone mineralization. Additionally, subsequent investigation of the level of mRNA expression revealed that the gamma-irradiated Ti surface facilitated as a great inducer in osteogenesis of hMSCs in most cases of the experiments. Although the gamma-irradiated Ti discs (10 kGy, 25 kGy) were able to enhance the osteoblastic activity and osteoblastic markers, the GA-modified Ti disc showed the lowest osteogenesis-inducing potential among the modified surfaces. These results showed that the osteoblastic differentiation of hMSCs can be altered by modifying the surface with gamma-irradiation. 

The in vivo measurement of bone volume gives information about the three-dimensional thickness of newly grown bone tissue around the implant, which correlates with BIC; therefore, bone volume differences are important when comparing impacts on different implant surfaces and bone formation [49]. The previous in vivo studies including the similar animal model with this study have proven the advantageous properties of collagen type I, which improve the peri-implant bone remodeling [11,15,50,51]. In the study by Schliephake et al. [51], the coating of Ti screws with collagen alone resulted in more increased bone growth around the implant and BIC than in implants with machined surfaces. Also, Sartori et al. [52] investigated the Ti implants functionalized with collagen in the healthy and osteopenic rat model and reported the results that the collagen-treated implants promoted osteointegration in both healthy and compromised bone. Furthermore, they also evaluated the mechanical stability and ensured the greater mechanical stability in collagen treated screws with 23% higher value than untreated implants at 12 weeks post-implantation. In present results of bone volume and BIC have shown that the treatment of collagen on the implant surfaces resulted in enhancement of osseointegration and bone remodeling than the untreated group. Furthermore, this result indicates that collagen, the principal organic matrix in bone, has positive effects on bone tissue around the implant.

The gamma-irradiated Ti have proved their potential to improve osteogenesis of hMSCs using an ALP activity assay and ARS staining, and it was demonstrated that the radiation technique on the titanium surface allowed for osteogenic gene expression in hMSCs. Furthermore, through the in vivo results in the rat tibia model, the Ti implants coated with collagen crosslinked using gamma irradiation was able to achieve sufficient bone formation accompanied by high bone and implant binding. However, the present study was performed using a small number of animals and observations at only 4 weeks after implantation; therefore, further larger-scale, more comprehensive studies with the large animal model should be needed. Furthermore, the additional studies are also required to optimize the radiation dose and collagen concentration to get a more ideal crosslinking result.

## 4. Materials and Methods

### 4.1. Preparation of Crosslinked Collagen Coated Ti Implants 

To prepare the collagen type I solution, collagen (source: porcine skin, atelocollagen type I, Matrixen-PSP, Sk Bioland Co. Ltd., Cheonan, Korea) was dissolved in 0.05 M acetic acid (Sigma-Aldrich) at room temperature (RT) to a final concentration of 0.5% (*w*/*v*). The titanium discs and implants (length: 2.5 mm, diameter: 1.5 mm, Dentium Co., Seoul, Korea) were placed in collagen solution in 10 mL glass vial for 24 h. To crosslink collagen onto titanium using chemical crosslinking agents (GA group), the samples were immersed in 2.5% (*v*/*v*) glutaraldehyde (GA) for 1 h at RT. The samples of the GA group were washed with distilled water (DW) for 48 h at RT to remove the unreacted collagen and glutaraldehyde. To prepare the 10 kGy and 25 kGy group, the collagen was crosslinked onto titanium surfaces using ^60^Co gamma-irradiation (MDS Nordion, Ottawa, ON, Canada, Korea Atomic Energy Institute) and a dose rate of 10 kGy/hr at RT. After irradiation, unreacted collagen or crosslinking agents were washed out via stirring with diH_2_O for 24 h. Collagen-coated titanium specimens were then dried for 3 days at RT in a vacuum oven.

### 4.2. In Vitro Analysis

#### 4.2.1. Scanning Electron Microscopy (SEM)

Scanning electron microscopy (SEM) was conducted to observe the surface of each group. The specimens were sputter-coated with gold using a sputter coater (SCD 005, BAL-TEC (Leica Microsystems GmbH), Wetzlar, Germany) and were observed using SEM (Hitachi S3500N, Hitachi, Japan) with an acceleration voltage of 15 kV.

#### 4.2.2. Trinitrobenzensulonic Acid (TNBS) Assay

The amount of collagen that was crosslinked on titanium implants was detected using a trinitrobenzensulonic acid (TNBS) assay of the un-crosslinked free amino groups of collagens after reaction with glutaraldehyde or gamma irradiation. Briefly, the collagen crosslinked on titanium discs were incubated with slow shaking in a 0.5% TNBS solution in a water bath and hydrolyzed with 6N HCl was complete at 90 min in the water bath. The absorbance of the diluted TNBS solution that reacted to samples were quantified by measuring at 349 nm using ultraviolet (UV)/visible spectroscopy (PowerWave XS, Bio-tek, Winooski, VT, USA).

#### 4.2.3. Cell Culture

Human mesenchymal stem cells (hMSCs) purchased from LONZA (Walkersville, MD, USA) were used for the in vitro study. The cells were cultured in an MSC growth medium (MSCGM; LONZA) for an expansion and for the examination of proliferation. The culture medium was changed every 3 days until confluence. The cells from the third to fourth passage were used for all the experiments. The differentiation of hMSCs into osteoblasts was induced using an osteogenic medium containing ascorbic acid-2-phosphate (Sigma-Aldrich, Milan, Italy), 10 mM β-glycerophosphate (Sigma-Aldrich) and 100 nM dexamethasone (Sigma-Aldrich) in an alpha-modification of Eagle’s medium (α-MEM; Welgene Inc., Deagu, Korea) supplemented with 10% fetal bovine serum (FBS; Gibco BRL, Carlsbad, CA, USA), 100 U/mL penicillin, and 100 μg/mL streptomycin (Gibco BRL).

#### 4.2.4. Cell Proliferation and Toxicity Assay

The proliferation and toxicity of hMSCs were evaluated by using a 3-[4,5-dimethylthiazol-2-yl]-2,5-diphenyl tetrazolium bromide (MTT; Sigma-Aldrich) assay. Ti discs were positioned on the bottom of a 48-well plate (Nunc, Roskilde, Denmark) and the cells (5 × 10^3^ cells/well) were seeded in the plates in triplicate in four different 48-well culture plates (Nunc, Roskilde, Denmark). After 0, 1, 2, and 3 days of incubation, the culture medium was replaced with a serum-free medium containing 30 μL/well of MTT solution (0.5 mg/mL MTT in PBS). After an additional 3 h of incubation in 5% CO_2_ at 37 °C, the culture medium with the MTT solution was discarded and 300 μL/well of dimethyl sulfoxide (DMSO; Duchefa Biochemie, Haarlem, Netherlands) was added to lyse formazan crystals. The plates were then agitated on a shaker for a couple of minutes to solubilize the formazan crystal. Two 100 μL/well aliquots were transferred to a 96-well plate (Nunc) and the absorbance values were measured at a wavelength of 570 nm with an Opsys MR microplate reader (DYNEX Technologies Inc., Denkendorf, Germany). The cell proliferation rate was shown in optical density (OD) units and normalized by the relative value of day 0. The absorbance data of day 1 were used to show the percentage of cytotoxicity and were normalized to the relative value of control.

#### 4.2.5. Alkaline Phosphatase (ALP) Activity Assay and Alizarin Red S (ARS) Staining

An ALP activity assay and ARS staining were used to evaluate the osteogenic differentiation of MSCs. Ti discs were positioned on the bottom of 48-well plates before cell seeding. The cells (5 × 10^3^ cells/well) were seeded in the 48-well plates in triplicate and cultured for 4 and 8 days in the osteogenic media. On day 4, the early stage of osteogenic differentiation was analyzed using a TRACP & ALP assay kit (Takara Bio Inc., Otsu, Shiga, Japan) according to the manufacturer’s protocol. After 4 days of incubation, the 100 μL medium was mixed with a 100 μL substrate solution containing 20 mmol/L Tris-HCl, 1 mmol/L MgCl2, and 12.5 mmol/L p-nitrophenyl phosphate (pNPP), and further incubated at 37 °C for 30 min in the dark. Using the addition of 100 μL NaOH (0.9 N), the reaction was stopped, and the absorbance rate was read at 405 nm using an Opsys MR microplate reader. On day 8, the late stage of osteogenic differentiation was determined using ARS staining for the detection of calcification. To assess the ARS staining, the cells were washed with PBS and fixed for 15 min with 3.7% formaldehyde. After being washed twice with distilled water, the fixed cells were stained with a 2% ARS solution (Sigma-Aldrich) for 30 min. Quantification of the staining images were done using using ImageJ software program (U.S. National Institutes of Health, Bethesda, MD, USA).

#### 4.2.6. Real-Time Polymerase Chain Reaction (PCR) Analysis

The real-time polymerase chain reaction (PCR) analysis was performed to examine the expression of osteogenic genes in hMSCs. Before cell seeding, Ti discs were positioned on the bottom of six-well plates (Nunc) and the cells (5 × 10^3^ cells/well) were seeded in the plates and cultured in osteogenic media for 4 and 8 days. Total RNAs were extracted from the cells cultured on the samples by using TRIzol (Life Technologies, Grand Island, NY, USA), and the RNA concentration was measured by using a NanoDrop ND-1000 spectrophotometer (Technologies Inc., Wilmington, DE, USA). The complementary DNAs (cDNAs) were synthesized by using 1 μg of total RNA. Amplification was done by using SYBR Green Master Mix reagents (Kapa Biosystems, Woburn, MA, USA) and an ABI 7500 instrument (Applied Biosystems, Carlsbad, CA, USA) according to the manufacturers’ protocols. Actin was used as an endogenous control and the relative mRNA expression levels of each gene were normalized by the mRNA expression of actin. The primers were synthesized and provided by Bionics (Daejeon, Korea) (Table 3).

### 4.3. In Vivo Analysis

#### 4.3.1. Experimental Animals

This research project was approved by the Institutional Animal Care and Use Committee of Pusan National University (PNU-2018-1977). Six Sprague Dawley rats (male, 12-week-old, KoaTech Inc., Gyunggi-do, Korea) weighing approximately 300 g were used in this experiment. The rats were adapted for a minimum of 7 days in the individual cages and were fed *ad libitum* rodent pellets and water. The laboratory was maintained standard laboratory conditions (temperature: 25 ± 1 °C, humidity: 55 ± 7%). All the surgical procedures were performed at the laboratory animal resource center. 

#### 4.3.2. Surgical Procedures

**Figure 9:**
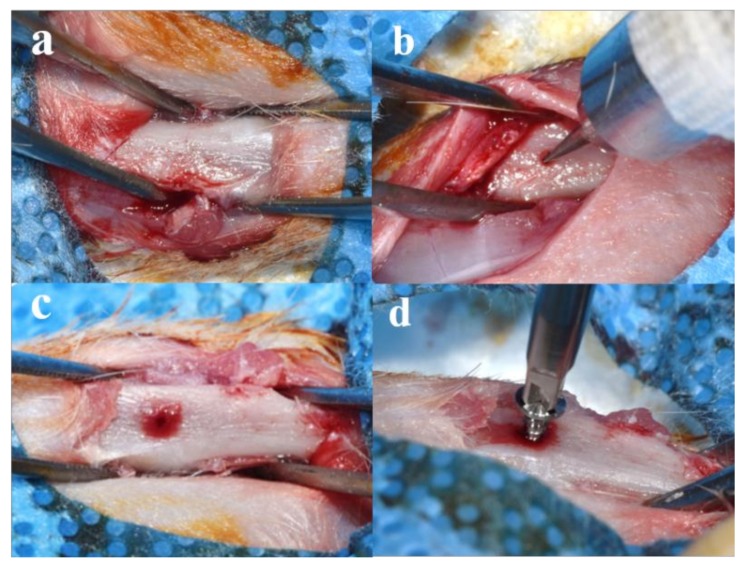
Surgical procedures for installation of collagen coated implant in rat tibia: (**a**) preparation of rat tibia for surgery; (**b**,**c**) drilling procedures; and (**d**) installation of a randomly assigned implant.

All the rats were anesthetized using intramuscular injection with a mixture of xylazine (Rompun, Bayer Korea, Seoul, Korea) and Tiletamine-zolazepam (Zoletil 50^®^, Virbac Laboratories, Carros, France) for general anesthesia. Surgical sites of rat were clearly shaved and disinfected with povidone-iodine (Betadine, Korea Pharma Co., Seoul, Korea). The local anesthesia was performed via injection of 2% lidocaine with 1:100,000 epinephrine (Yuhan, Seoul, Korea). After making an 2 mm incision on the tibia using surgical scalpel blade (No. 15, Swann-Morton Ltd., Sheffield, England), skin and muscle were elevated, and the tibia was exposed (Figure 9a). A hole for implant installation was made using a dental carbide bur (round, #4, diameter 1.4 mm, Dentsply Professional Division, York, PA, USA) while irrigating with saline (Figure 9b,c). Each side of the tibia was randomly assigned to the three study groups (control, GA, and 25 kGy groups) and implants of each group were installed into tibia using a hand driver (Dentium Co., Seoul, Korea) (Figure 9d). The skin was sutured with absorbable sutures (Vicryl^®^, 4-0, Ethicon, NJ, USA). Animals were euthanized 4 weeks after surgery using CO_2_ inhalation. Surgical sites were harvested, and skin and muscle were carefully removed. The collected samples were fixed in neutral buffered formalin (Sigma Aldrich Co., St. Louis, MO, USA) for 2 weeks.

#### 4.3.3. Micro-Computed Tomography (μCT) Analysis

**Figure 10:**
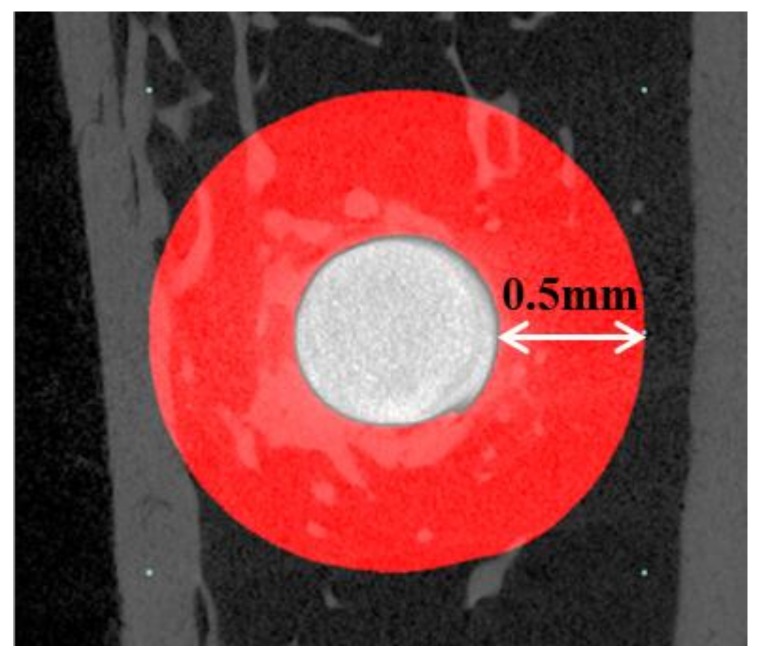
The image section of micro-computed tomography (μCT). White circle: a head of installed implant; Red region: region of interest (ROI) of bone volume measurement.

The collected tibia specimens were wrapped with Parafilm (Bemis Flexible Packaging, Neenah, WI, USA) to prevent fixative solution evaporation during scans. μCT scanning was conducted using a μCT scanner (Skyscan-1173, ver. 1.6, Bruker-CT Co., Kontich, Belgium) under the following conditions: resolution—13.85 μm, source voltage—130 kV, source current—60 μA, image pixel size—6.04 μm, exposure—500 ms, and rotation step—0.3°. The Nrecon reconstruction program (Nrecon, ver. 1.7.0.4, Bruker-CT Co., Kontich, Belgium) was used to reconstruct images. A region of interest (ROI) was 0.5 mm in width around the implant (Figure 10).

#### 4.3.4. Histology Analysis

After the μCT analysis was completed, the specimens were dehydrated using an alcohol series with increasing concentrations (70, 80%, 90%, and 100% ethanol). For the resin infiltration, the specimens were infiltrated using a mixture of Technovit 7200 resin (Heraeus KULZER, Hanau, Germany)/ethanol with increasing resin ratios, and then infiltrated with pure Technovit 7200 resin for a week. For embedding, the infiltrated specimens were fixed using the embedding frame with the implanted site was facing downwards. The embedding frame was cured using a UV embedding system (KULZER EXAKT 520, Heraeus Kulzer, Germany) for 24 h. The polymerized blocks were sectioned to a thickness of 400 μm at the center of the implant using the diamond cutter (KULZER EXAKT 300 CP Band System, Exakt Apparatebau, Norderstedt, Germany). Using the EXAKT grinding machine (KULZER EXAKT 400CS, Exakt Apparatebau, Norderstedt, Germany), the sections were polished to a thickness of 30 μm, and then mounted on slides stained with H&E stain.

**Figure 11:**
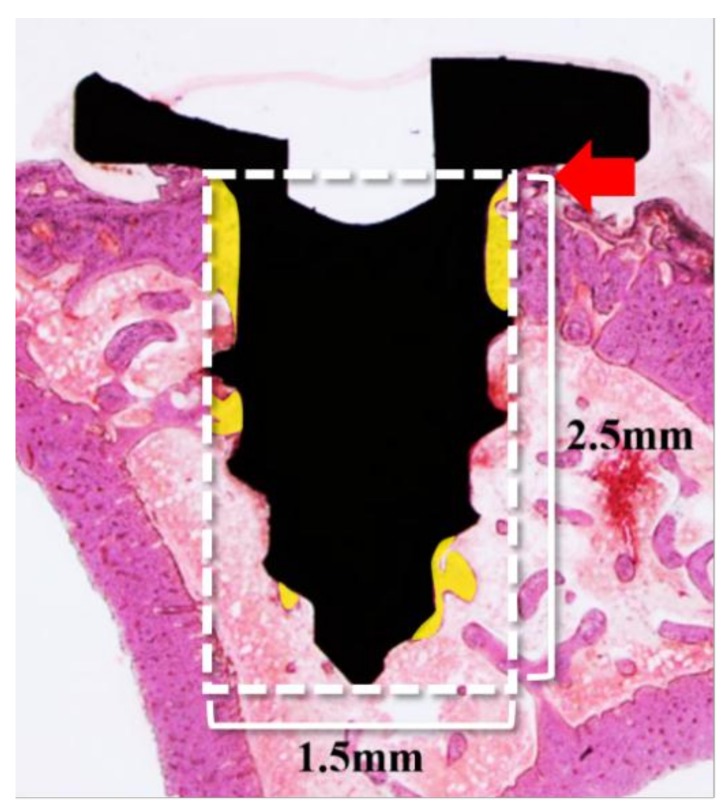
Parameters measured in the bone-to-implant contact (BIC) in histologic analysis. White box; Region of interest (ROI). Red arrow: the end of the implant head; Yellow area: calculated region within ROI.

The final slides were observed and imaged using a light microscope (Olympus BX51, Olympus Optical Co., Tokyo, Japan) connected with a couple-charged device (CCD) camera (SPOT Insight, Scientific Co. Inc., Campbell, CA, USA), and an adaptor (U-CMA3, Olympus Co., Nagano, Japan). Bone-to-implant contact (BIC) was measured using the image analysis program (i-solution, IMT, Vancouver, BC, Canada) using a single, professionally trained and blinded investigator. The region of interest (ROI) of BIC was 1.5 mm in width and 2.5 mm in height from the end of the implant head to the apex (Figure 11).

### 4.4. Statistical Analysis

In vitro results were analyzed using one-way analysis of variance (ANOVA) and Tukey’s post-hoc test using SPSS statistical analysis software (ver. 23.0, IBM Corp, Armonk, NY, USA). In vivo results were analyzed using Brunner and Langer’s method [53] using statistical software R (ver. 3.2.5, The R Foundation, Vienna, Austria). The statistical analysis was performed with a confidence level of 95% (*p* < 0.05).

## 5. Conclusions

The present study was conducted to evaluate the titanium implants coated with collagen type I crosslinked by gamma irradiation to enhance the osseointegration. The in vitro results revealed higher cell proliferation and osteogenic differentiation in the specimen using gamma irradiation without cytotoxicity compared with specimen using the chemical crosslinking, and the in vivo results indicated that the collagen-treated Ti implants using radiation showed fine osseointegration. Within the limitations of this study, Ti implant coated with radiation-crosslinked collagen has potential utility without the adverse effects from chemical agents.

## Figures and Tables

**Table 1:** New bone volume within regions of interest (mm^3^)

Group	Mean ± SD	Median
Control	5.55 ± 0.48	5.55
GA	7.50 ± 1.14	7.81
25 kGy	6.87 ± 2.01	7.47
*p*-value	0.006 **	

The symbol “*” indicates statistical significance between the three groups (** *p* < 0.01).

**Table 2:** Bone-to-implant contact within regions of interest (BIC, %).

Group	Mean ± SD	Median
Control	35.21 ± 3.98	35.60
GA	49.66 ± 13.84	54.22
25 kGy	9.66 ± 10.47	50.19
*p*-value	0.019 *	

The symbol “*” indicates statistical significance between the three groups (* *p* < 0.05).

**Table 3:** Primer sequences used for real-time PCR analysis.

Target Genes	*Sequences*
*Runx2*	F: 5′-TGCTTTGGTCTTGAAATCACA-3′
	R: 5′- TCTTAGAACAAATTCTGCCCTTT-3′
*BMP-2*	F: 5′-AACACTGTGCGCAGCTTCC-3′
	R: 5′-CTCCGGGTTGTTTTCCCAC-3′
*ALP*	F: 5′-ATTTCTCTTGGGCAGGCAGAGAGT-3′
	R: 5′-ATCCAGAATGTTCCACGGAGGCTT-3′
*OCN*	F: 5′-CAGCGAGGTAGTGAAGAGAC-3′
	R: 5′-TGAAAGCCGATGTGGTCAG-3′
*OPN*	F: 5′-AGACACATATGATGGCCGAGG-3′
	R: 5′-GGCCTTGTATGCACCATTCAA-3′
*Actin*	F: 5′-ACTCTTCCAGCCTTCCTTCC-3′
	R: 5′-TGTTGGCGTACAGGTCTTTG-3′

## Abbreviations

BIC	Bone-to-implant contact
Ti	Titanium
GA	Glutaraldehyde
ROI	Region of interest
SEM	Scanning electron microscopic
ALP	Alkaline phosphatase
ARS	Alizarin Red S
Real-time PCR	Real-time polymerase chain reaction
H&E	Hematoxylin-eosin
MTT	3-[45-dimethylthiazol-2-yl]-2,5-diphenyl tetrazolium bromide
μCT	Micro-computed tomography
ANOVA	Analysis of variance
RT	Room temperature
CMC	Carboxymethyl cellulose
MC	Methylcellulose
